# Radiomics Signatures of Carotid Plaque on Computed Tomography Angiography: An Approach to Identify Symptomatic Plaques

**DOI:** 10.1007/s00062-023-01289-9

**Published:** 2023-05-17

**Authors:** Jinglong Shi, Yu Sun, Jie Hou, Xiaogang Li, Jitao Fan, Libo Zhang, Rongrong Zhang, Hongrui You, Zhenguo Wang, Anxiaonan Zhang, Jianhua Zhang, Qiuyue Jin, Lianlian Zhao, Benqiang Yang

**Affiliations:** 1 Jinzhou Medical University General Hospital of Northern Theater, Command Postgraduate Training Base, Shenyang, China; 2 Department of Radiology, General Hospital of Northern Theater Command, 83 Wenhua Road, 110016 Shenyang, Liaoning Province China; 3 Key Laboratory of Cardiovascular Imaging and Research of Liaoning Province, Shenyang, China; 4 Beijing Deepwise & League of PHD Technology Co. Ltd, Beijing, China

**Keywords:** Carotid atherosclerosis, Stroke, Plaque vulnerability, Nomogram, Machine learning

## Abstract

**Purpose:**

To develop and validate a combined model incorporating conventional clinical and imaging characteristics and radiomics signatures based on head and neck computed tomography angiography (CTA) to assess plaque vulnerability.

**Methods:**

We retrospectively analyzed 167 patients with carotid atherosclerosis who underwent head and neck CTA and brain magnetic resonance imaging (MRI) within 1 month. Clinical risk factors and conventional plaque characteristics were evaluated, and radiomic features were extracted from the carotid plaques. The conventional, radiomics and combined models were developed using fivefold cross-validation. Model performance was evaluated using receiver operating characteristic (ROC), calibration, and decision curve analyses.

**Results:**

Patients were divided into symptomatic (*n* = 70) and asymptomatic (*n* = 97) groups based on MRI results. Homocysteine (odds ratio, OR 1.057; 95% confidence interval, CI 1.001–1.116), plaque ulceration (OR 6.106; 95% CI 1.933–19.287), and carotid rim sign (OR 3.285; 95% CI 1.203–8.969) were independently associated with symptomatic status and were used to construct the conventional model and s radiomic features were retained to establish the radiomics model. Radiomics scores incorporated with conventional characteristics were used to establish the combined model. The area under the ROC curve (AUC) of the combined model was 0.832, which outperformed the conventional (AUC = 0.767) and radiomics (AUC = 0.797) models. Calibration and decision curves analysis showed that the combined model was clinically useful.

**Conclusion:**

Radiomics signatures of carotid plaque on CTA can well predict plaque vulnerability, which may provide additional value to identify high-risk patients and improve outcomes.

**Supplementary Information:**

The online version of this article (10.1007/s00062-023-01289-9) contains supplementary material, which is available to authorized users.

## Introduction

Stroke is a leading cause of death and disability, with a majority of ischemic stroke of all stroke types [1]. Carotid atherosclerosis accounts for approximately 18–25% of ischemic strokes [2]. High-resolution magnetic resonance imaging (MRI) can detect carotid plaque characteristics, including intraplaque hemorrhage (IPH), lipid-rich necrotic cores, and fibrous caps, which have been shown to be associated with plaque vulnerability [3]; however, due to the long acquisition time and patient contraindications of carotid MRI, acute stroke decision-making is often based on computed tomography (CT) and CT angiography (CTA) as a quick, easy and widely used imaging modality to identify cerebral hemorrhage or artery occlusion [4]. For patients with suspected carotid atherosclerosis, CTA provides a substitute for patients without access to MRI, and therefore a better understanding of carotid plaque characteristics on CTA is imperative.

Although the measurement of carotid artery stenosis is an important means to stratify patients with treatment, other plaque characteristics on CTA are helpful in estimating plaque vulnerability [5]. Plaque features, such as length [6], thickness [7], ulceration [8], and texture [4, 9] are substantially related to the onset of ischemic stroke. Furthermore, a large-sample study (*n* = 19,804) conducted by Martins et al. found that morphological abnormalities of the internal carotid artery (ICA) were associated with hypertension, hyperlipidemia, diabetes, and heart disease, and ICA tortuosity was associated with ipsilateral cerebral ischemia [10]. Therefore, plaque composition and biomechanics were critical factors for carotid artery plaque risk assessment [11].

Compared with conventional assessment, radiomics provides more comprehensive information by converting images to higher dimensional data and allowing high-throughput extraction of quantitative image features to provide decision support and accurately phenotype diseases in vivo [12–15]. Previous studies revealed that radiomic features improved the ability to detect vulnerable plaques on coronary CTA [16, 17] and identify symptomatic plaques in the carotid [18] and basilar arteries [19] on MRI. Recently, CTA-based radiomics analysis also showed promising results in carotid plaques analysis [20]. In this study, we aimed to investigate conventional and radiomic features of carotid atherosclerotic plaques on CTA associated with symptomatic plaque status. We also integrated critical characteristics to build a high-risk plaque model and evaluate its performance in differentiating symptomatic from asymptomatic carotid plaques.

## Material and Methods

### Study Population

**Fig. 1:**
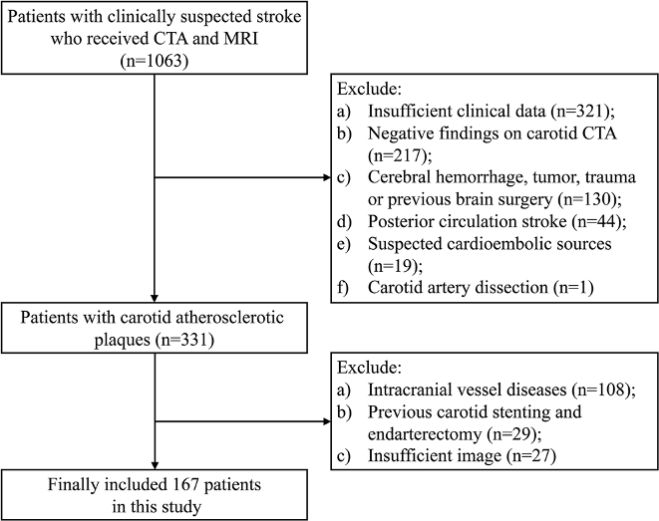
A flowchart of patient recruitment. *CTA* computed tomography angiography, *MRI* magnetic resonance imaging

This retrospective study was approved by the local institutional review committee (No. Y [2022]114), and the requirement for informed consent was waived. We included 1063 consecutive patients with clinically suspected stroke who underwent head and neck CTA and brain MRI within 1 month between October 2019 and July 2022. The exclusion criteria were as follows: (1) insufficient clinical data; (2) negative findings on carotid CTA; (3) presence of cerebral hemorrhage, tumor, trauma or previous brain surgery; (4) posterior circulation stroke; (5) suspected cardioembolic sources; (6) carotid artery dissection; (7) accompanied by intracranial vessel diseases, such as atherosclerotic stenosis, aneurysm or moyamoya disease; (8) previous carotid stenting and endarterectomy; (9) insufficient image quality for plaque analysis and radiomics extraction (see Fig. 1 for the flow chart of patient selection).

Demographic, clinical and laboratory data were collected from all patients, including age, sex, smoking status, hypertension, diabetes mellitus, hyperlipidemia, coronary artery disease, medication history, total cholesterol, triglycerides, high-density lipoprotein cholesterol, low-density lipoprotein cholesterol, lipoprotein(a) and homocysteine (Hcy).

### Imaging Acquisition

Head and neck CTA was performed using a 256-slice CT scanner (Brilliance iCT; Philips Medical Systems, Cleveland, OH, USA). The aortic arch to the skull vertex was covered by CTA examination in the helical scanning mode. The scanning parameters were as follows: detector collimation, 128 × 0.625 mm; tube voltage, 120 kV; tube current, 250 mA; rotation time, 0.33 s; pitch, 0.763; section increment, 0.45 mm; and slice thickness, 0.9 mm. Approximately 50–60 mL of nonionic iodine contrast agent was injected at a flow rate of 4.0–5.0 mL/s via a high-pressure injector, followed by a 20–30 mL flush of saline at the same rate.

The MRI examinations were performed on a 3.0T MR scanner (Discovery 750; GE Healthcare, Chicago, IL, USA) with a 20-channel head coil using standardized protocols, including T1 and T2 weighted imaging, T2 fluid-attenuated inversion recovery (FLAIR) and diffusion weighted imaging (DWI). Ischemic lesions were assessed on DWI with the following parameters: TR 4000 ms, TE 68 ms, FOV 24 cm×24 cm, slice thickness 6 mm, matrix 128 × 128, intersection gap 1.2 mm, number of excitations 1, b‑values of 0 and 1000 s/mm^2^.

### Imaging Analysis

**Fig. 2:**
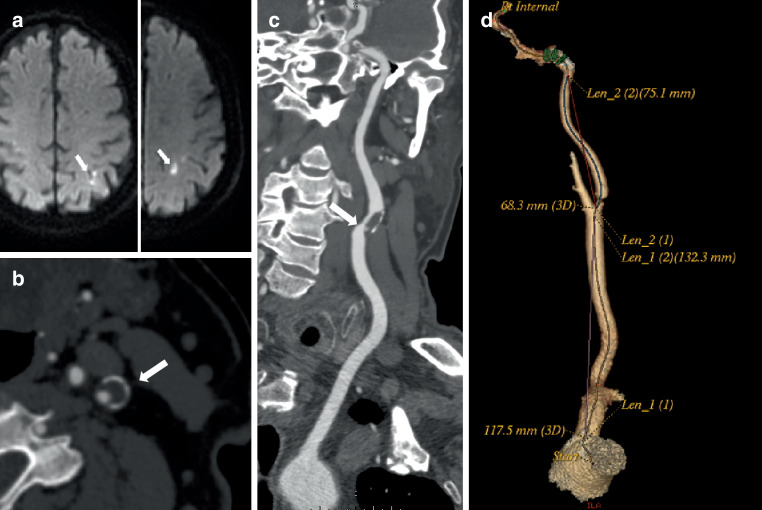
Carotid computed tomography angiography (CTA) imaging markers and stroke work-up. This 73-year-old man presented with abrupt onset right-sided weakness and numbness. Axial diffusion-weighted imaging (**a**) showed an acute infarct (*long arrows*). Axial CTA of the carotid arteries (**b**) demonstrated a thick left proximal internal carotid artery plaque with a positive rim sign (*long arrow*). Multiplanar reformat of CTA images (**c**) demonstrated the ulcerated carotid plaque (*long arrow*). Three-dimensional volume rendering (**d**) showed the linear distance of the vessel was determined as the shortest distance between the same two points, the actual distance of the vessel was measured from the proximal to distal point along the arterial center line from the curved projection reformation

Carotid plaque characteristics were assessed on CTA using GE Healthcare Advantage Workstation (AW4.4, USA). Carotid stenosis, plaque features and carotid artery tortuosity were measured and evaluated as follows: (1) the degree of carotid stenosis was defined as mild (< 50%) and moderate to severe (50–99.9%) stenosis using the North American Symptomatic Carotid Endarterectomy Trial (NASCET) criteria [21]; (2) the maximum plaque thickness: measured on the cross-section of the plaque perpendicular to the long axis of the vessel, (3) the soft plaque thickness: a plaque with a mean attenuation of 40–50 HU was considered soft [5]; (4) plaque ulceration: defined as at least 1 mm of contrast agent entering the plaque in any one plane [22]; (5) carotid rim sign: a positive carotid rim sign was defined as adventitial calcification (< 2 mm thickness) with interior soft plaque (> 2 mm thickness), whereas a negative carotid rim sign was defined as adventitial calcification (< 2 mm thickness) with little interior soft plaque (< 2 mm thickness) ([23]; Fig. 2); (6) positive remodelling (PR): characterized as an outside vessel diameter that was 10% larger than the mean diameter of the segments that were both proximal and distal to the plaque [24] and (7) common carotid tortuosity index (CCTI) and internal carotid tortuosity index (ICTI): calculated using the following formula: ([actual distance/linear distance] − 1) × 100 [25]. The actual distance of the vessel was measured along the arterial center line from the proximal to the distal point in the curved projection reformation. The linear distance of the vessel was determined as the shortest distance between the same two points in the three-dimensional volume rendering image. Proximal vessel points were uniformly placed at the arterial origin. The common carotid artery was placed distally at the carotid bifurcation, and the ICA was placed distally at the entrance of the outer carotid canal (Fig. 2).

Patients were divided into symptomatic and asymptomatic groups according to the following criteria: (1) clinical symptoms continuing for at least 24 h within 2 weeks before imaging scans; and (2) acute/subacute stroke findings on DWI in the ipsilateral carotid artery territory [26, 27].

### Radiomics Plaque Segmentation

Patient data were anonymized and transmitted to The Deepwise Multimodal Research Platform v2.0 (Beijing Deepwise & League of PHD Technology Co., Ltd, Beijing, China, https://keyan.deepwise.com), which can be used for labeling, region of interest (ROI) feature extraction, and model development [20, 28]. In the symptomatic group, we segmented the carotid plaque located ipsilateral to the infarct. For patients with bilateral plaques in the asymptomatic group, we segmented unilateral plaque with a larger volume. For patients with plaques involving both the internal and external carotid arteries, we only segmented the internal carotid plaques. A radiologist visually assessed the outer border of the arterial wall and segmented the plaque boundaries with an appropriate center level and window width.

### Reproducibility Evaluation

In a subgroup of patients (*n* = 30) who were randomly chosen from the research population, two radiologists independently segmented the plaques for radiomic features extraction and assessed the conventional features 2 weeks after the first assessment. Intraclass correlation coefficient (ICC) and Cohen’s kappa coefficient were calculated for the intraobserver and interobserver consistency: < 0.40, poor agreement; 0.41–0.60, moderate; 0.61–0.80, substantial; and > 0.80, excellent.

### Model Development

**Fig. 3:**
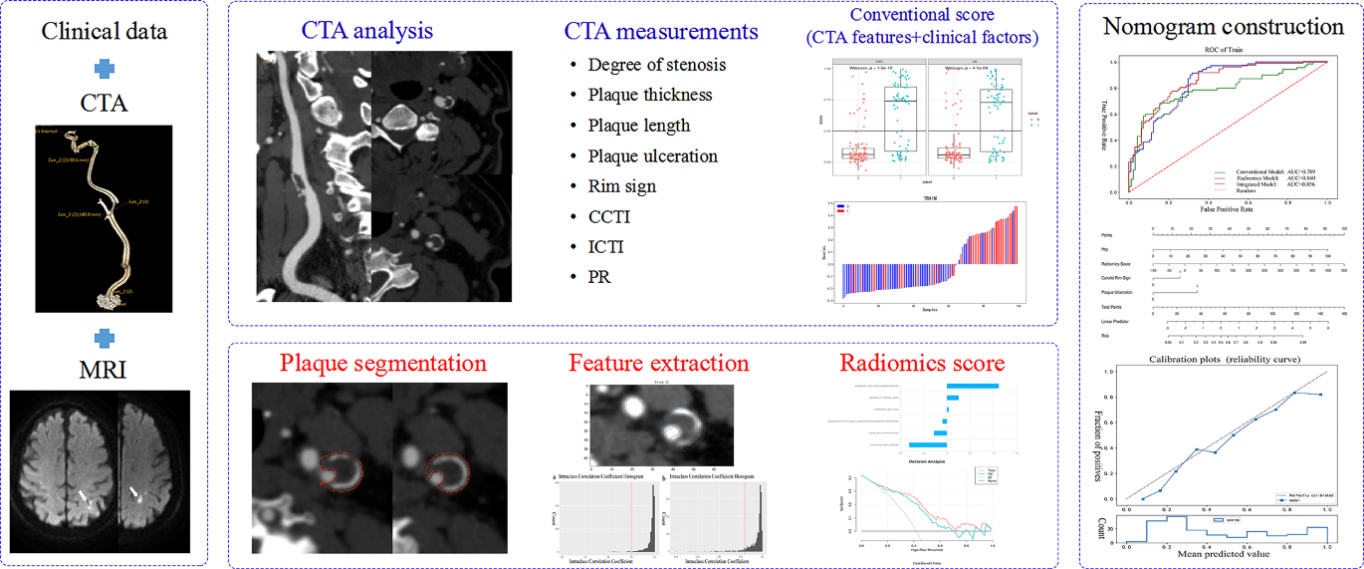
A flow chart displaying the process for development of radiomics based integrated score. *CTA* computed tomography angiography, *MRI* magnetic resonance imaging, *CCTI* common carotid tortuosity index, *ICTI* internal carotid tortuosity index, *PR* positive remodelling

We selected clinical and conventional plaque characteristics with statistical differences in univariate and multivariable logistic regression analyses and built the conventional model. For radiomics model development, we first preserved radiomic features with intraobserver and interobserver ICC > 0.8 and eliminated redundant features with linear correlation coefficient values (threshold = 0.7). We then used the analysis of variance F‑value, mutual information, and linear models penalized with the L1 norm algorithm to form radiomics signature. Finally, a combined model was created by integrating selected conventional characteristics and radiomics signature. All models were trained using the fivefold cross-validation method. The workflow of the model development is shown in Fig. 3.

## Statistical Analysis

All statistical analyses were performed using SPSS software (SPSS statistics, version 25.0; SPSS Inc., Armonk, NY, USA: IBM Corp.), statistical software packages R (version 3.3.2; http://www.R-project.org, The R Foundation), and Free Statistics software (version 1.7). The consistency of ROI segmenting, dimension reduction of features and ML model development were performed on the Deepwise Multimodal Research Platform v2.0. Categorical variables are reported as frequencies (%) and continuous variables as means ± standard deviation or median (interquartile range). Classified variables were compared using the χ^2^-test. Comparisons of continuous variables were performed using Student’s *t*-test or Mann–Whitney *U* test. Variables with statistical significance in the univariate analysis were used in the multivariate logistic analysis. Model performance was evaluated using receiver operating characteristic (ROC), calibration, and decision curve analyses. All statistical tests were two-sided. Statistical significance was set at *p* < 0.05.

## Results

### Clinical Characteristics

In total, 167 patients were included in the final analysis. Patients were mainly older (66.2 ± 7.7 years; 78.4% male), among whom smoking (54.5%) and hypertension (68.9%) accounted for a higher proportion. The clinical characteristics of patients in the symptomatic and asymptomatic groups are shown in Table 1. Smoking was more prevalent in the symptomatic group than in the asymptomatic group (65.7% vs. 46.4%, *p* = 0.013). Total cholesterol (4.7 vs. 4.2 mmol/L, *p* = 0.022), lipoprotein(a) (231.4 vs. 134.2 mg/L, *p* < 0.002), and Hcy (13.9 vs. 11.0 µmol/L, *p* < 0.001) were higher in the symptomatic group than the asymptomatic group. Multivariate logistic regression analysis revealed that Hcy (odds ratio, OR, 1.057; 95% confidence interval, CI, 1.001–1.116) was the only independent risk factor associated with symptomatic plaques (Table 2).

**Table 1:** Imaging and clinical characteristics of patients

Variables	Total *n* = 167	Asymptomatic *n* = 97	Symptomatic *n* = 70	*p*
Age (years, mean ± SD)	66.2 ± 7.7	65.2 ± 7.7	67.6 ± 7.6	0.056
Male sex, *n* (%)	131 (78.4)	73 (75.3)	58 (82.9)	0.239
Smoking, *n* (%)	91 (54.5)	45 (46.4)	46 (65.7)	0.013^*^
Hypertension, *n* (%)	115 (68.9)	64 (66)	51 (72.9)	0.344
Diabetes mellitus, *n* (%)	48 (28.7)	33 (34)	15 (21.4)	0.076
Hyperlipidemia, *n* (%)	73 (43.7)	40 (41.2)	33 (47.1)	0.448
CAD, *n* (%)	23 (13.8)	11 (11.3)	12 (17.1)	0.283
Antihypertension, *n* (%)	109 (65.3)	58 (59.8)	51 (72.9)	0.080
Statin, *n* (%)	25 (15.0)	13 (13.4)	12 (17.1)	0.504
Antiplatelet, *n* (%)	36 (21.6)	20 (20.6)	16 (22.9)	0.729
Days between MRI and CTA, median (IQR)	1 (0, 4)	1 (0, 3)	2 (0, 4)	0.161
TC, mmol/L, median (IQR)	4.3 (3.6, 5.1)	4.2 (3.4, 4.9)	4.7 (3.9, 5.4)	0.022^*^
TG, mmol/L, median (IQR)	1.4 (1.0, 2.0)	1.5 (1.0, 2.2)	1.4 (1.0, 1.7)	0.468
HDL‑C, mmol/L, median (IQR)	1.1 (0.9, 1.3)	1.1 (0.9, 1.3)	1.1 (0.9, 1.3)	0.298
LDL‑C, mmol/L, median (IQR)	2.4 (1.8, 3.0)	2.2 (1.8, 2.9)	2.7 (2.0, 3.2)	0.068
Lp(a), mg/L, median (IQR)	184.3 (62.9, 295.1)	134.2 (57.4, 248.9)	231.4 (97.9, 367.4)	0.002^*^
Hcy, µmol/L, median (IQR)	11.7 (9.6, 15.4)	11.0 (9.0, 12.5)	13.9 (10.4, 20.7)	< 0.001^*^
Degree of stenosis, %, mean ± SD	48.2 ± 18.3	44.9 ± 17.2	52.8 ± 18.9	0.006^*^
Mild stenosis (0–49.9%), *n* (%)	91 (54.5)	59 (60.8)	32 (45.7)	0.053
Moderate to severe stenosis (50–99.9%), *n* (%)	76 (45.5)	38 (39.2)	38 (54.3)	0.053
CCTI, median (IQR)	10.2 (5.9, 16.2)	10.0 (5.9, 15.2)	11.4 (5.9, 17.9)	0.314
ICTI, median (IQR)	13.0 (8.2, 20.9)	12.3 (8.1, 20.1)	14.3 (9.0, 21.2)	0.379
Carotid maximum total plaque thickness, mm, median (IQR)	4.6 (3.6, 5.5)	4.2 (3.5, 5.1)	4.9 (3.9, 6.2)	0.002^*^
Maximum soft plaque thickness, mm, median (IQR)	3.7 (2.5, 4.9)	3.2 (2.3, 4.3)	4.5 (3.5, 5.7)	< 0.001^*^
Carotid plaque length, mm, mean ± SD	23.0 ± 7.9	21.6 ± 7.7	24.9 ± 7.9	0.007^*^
Carotid plaque ulceration, *n* (%)	28 (16.8)	5 (5.2)	23 (32.9)	< 0.001^*^
Carotid rim sign, *n* (%)	34 (20.4)	8 (8.2)	26 (37.1)	< 0.001^*^
PR, *n* (%)	74 (44.3)	36 (37.1)	38 (54.3)	0.028^*^

Continues variables are presented as means ± standard deviation or median (interquartile range). Categorical variables are presented as *n* (%)

*SD* standard deviation, *IQR* interquartile range, *CAD* coronary artery disease, *TC* total cholesterol, *TG* triglycerides, *HDL‑C* high-density lipoprotein cholesterol, *LDL‑C* low-density lipoprotein cholesterol, *Lp(a)* lipoprotein (a), *Hcy* homocysteine, *CCTI* common carotid tortuosity index, *ICTI* internal carotid tortuosity index, *PR* positive remodelling

^*^ Indicated* p* < 0.05 with significance

**Table 2:** Univariate analysis and multivariable analysis of conventional factors

Variable	Univariate analysis		Multivariable analysis	
	OR 95% CI	*p*	OR 95% CI	*p*
Smoking	2.21 (1.17–4.18)	0.014	–	–
TC	1.21 (0.96–1.52)	0.102	–	–
LP(a)	7.33 (1.74–30.91)	0.007	–	–
Hcy	1.08 (1.03–1.14)	0.004	1.057(1.001–1.116)	0.045^*^
Degree of stenosis	1.02 (1.01–1.04)	0.007	–	–
Carotid maximum total plaque thickness	1.44 (1.16–1.79)	0.001	–	–
Maximum soft plaque thickness	1.64 (1.31–2.04)	< 0.001	–	–
Carotid plaque length	1.06 (1.01–1.10)	0.009	–	–
Carotid plaque ulceration	9.00 (3.22–25.20)	< 0.001	6.106 (1.933–19.287)	0.002^*^
Carotid rim sign	6.57 (2.75–15.7)	< 0.001	3.285 (1.203–8.969)	0.020^*^
PR	2.01 (1.08–3.76)	0.028	–	–

*TC* total cholesterol, *Lp(a)* lipoprotein (a), *Hcy* homocysteine, *PR* positive remodelling, *OR* odds ratio, *CI* confidence interval

^*^ Indicated *p* < 0.05 with significance

### Plaque Features on CTA

Univariate analysis showed that the degree of carotid artery stenosis (OR 1.02; 95% CI 1.01–1.04), total plaque thickness (OR 1.44; 95% CI 1.16–1.79), soft plaque thickness (OR 1.64; 95% CI 1.31–2.04), plaque length (OR 1.06; 95% CI 1.01–1.10), plaque ulceration (OR 9.00; 95% CI 3.22–25.20), carotid rim sign (OR 6.57; 95% CI 2.75–15.7), and PR (OR 2.01; 95% CI 1.08–3.76) were related to plaque vulnerability. Further multivariate logistic regression analysis revealed that plaque ulceration (OR 6.106; 95% CI 1.933–19.287) and carotid rim sign (OR 3.285; 95% CI 1.203–8.969) were independent predictors of symptomatic plaques (Table 2). Intraobserver and interobserver reproducibility of conventional imaging features assessment was excellent (supplementary materials Table S1).

### Radiomics Signature

A total of 2107 radiomic features were extracted and normalized using the Z‑score from each ROI. The ICC for calculating radiomic features is summarized in the supplementary material (Fig. S1). First, radiomic features with ICC ≤ 0.8 were removed by interobserver and intraobserver agreement, and the remaining 1916 features were included in the analysis. Another 112 features were removed because of low discrimination. Then 1760 features were removed according to the linear correlation value (threshold = 0.7). A mutual information algorithm was used to analyze all remaining features. Finally, six significant radiomic features were retained to construct the radiomics model by using the logistic regression algorithm provided by the Deepwise Multimodal Research Platform [20], including wavelet-HLL_gldm_GrayLevelNonUniformity, wavelet-LLH_firstorder_Mean, wavelet-HLL_glcm_Imc2, log-sigma-2-0-mm-3D_gldm_LargeDependenceLowGrayLevelEmphasis, square_glcm_InverseVariance, and the exponential_ngtdm_Strength.

### Model Development and Performance

We developed three formulas based on independent risk factors and radiomics signature to calculate each patient’s risk score. They were presented as follows:

$$\text{Risk score for the conventional model }=0.0421+0.7469\times \text{plaque ulceration }+0.6811\times \text{carotid rim sign }+0.6088\times \mathrm{Hcy};$$

$$\text{Risk score for the radiomics model }=-0.0584+(0.2886\times \text{wavelet-LLH}\_ \text{firstorder}\_ \text{Mean})+(1.1835\times \text{wavelet-HLL}\_ \text{gldm}\_ \text{GrayLevelNonUniformity})+(0.0687\times \text{wavelet-HLL}\_ \text{glcm}\_ \mathrm{Imc}2)+(-0.8601\times \text{ exponential}\_ \text{ngtdm}\_ \text{Strength})+(-0.2867\times \text{ square}\_ \text{glcm}\_ \text{InverseVariance})+(-0.1182\times \text{log-sigma-2-0-mm-3D}\_ \text{gldm}\_ \text{LargeDependenceLowGrayLevelEmphasis});$$

$$\text{Risk score for the combined model }=0.0644+1.1187\times \text{radiomics score }+0.5774\times \text{plaque ulceration }+0.3697\times \text{carotid rim sign }+0.5981\times \mathrm{Hcy}.$$

**Fig. 4:**
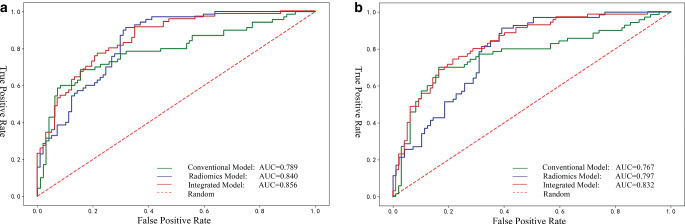
The ROC curve of the conventional, radiomics, and integrated models of symptomatic plaques in training (**a**) and validation cohort (**b**)

Table 3 shows the discriminative power of the three models. The radiomics model demonstrated a good diagnostic performance in the training (area under the curve, AUC 0.840; 95% CI 0.781–0.898) and validation cohorts (AUC 0.797; 95% CI 0.731–0.863). The conventional model had lower discrimination for symptomatic plaques in the training (AUC 0.789; 95% CI 0.715–0.863) and validation cohorts (AUC 0.767; 95% CI 0.689–0.845). The AUCs of the integrated model were the highest, with 0.856 (95% CI 0.800–0.913) and 0.832 (95% CI: 0.770–0.894) for the training and validation sets, respectively (Fig. 4). DeLong’s test showed that the integrated models significantly improved model performance compared to the conventional model in training (AUC 0.856 vs. 0.789,* p* = 0.005) and validation cohorts (AUC 0.832 vs. 0.767,* p* = 0.011). Although the diagnostic efficiency of the integrated model relatively outperformed the radiomics model, there was no significant difference in the training (AUC 0.856 vs. 0.840; *p* = 0.688) and validation cohorts (AUC 0.832 vs. 0.797; *p* = 0.452).

**Table 3:** Comparison of AUCs between the conventional, radiomics, and integrated models

Model	Training cohort				Validation cohort			
	SEN	SPE	ACC	AUC (95% CI)	SEN	SPE	ACC	AUC (95% CI)
Conventional model	0.629	0.856	0.761	0.789(0.715–0.863)	0.614	0.856	0.755	0.767(0.689–0.845)
Radiomics model	0.729	0.732	0.731	0.840(0.781–0.898)	0.686	0.711	0.701	0.797(0.731–0.863)
Integrated model	0.729	0.825	0.784	0.856(0.800–0.913)	0.700	0.804	0.761	0.832(0.770–0.894)

*SEN* sensitivity, *SPE* specificity, *ACC* accuracy, *AUC* area under receiver operating characteristic curve, *CI* confidence interval

### Nomogram Construction and Evaluation

**Fig. 5:**
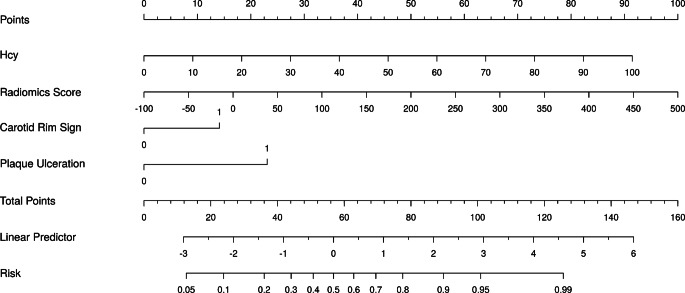
An integrated model nomogram was developed with homocysteine (Hcy), carotid rim sign, plaque ulceration and radiomics score of the selected radiomic features incorporated. Use the constructed nomogram to identify symptomatic plaques, carotid rim sign and plaque ulceration (0 indicates absent; 1 present)

On the basis of the combined model, a nomogram was constructed that incorporated three selected conventional features (Hcy, plaque ulceration, and carotid rim sign) and radiomics score for predicting plaque vulnerability (Fig. 5). The value of each of these variables was given a score on the point scale axis. A total score could be easily calculated by adding each single score to the total point scale to estimate the risk of plaque vulnerability.

**Fig. 6:**
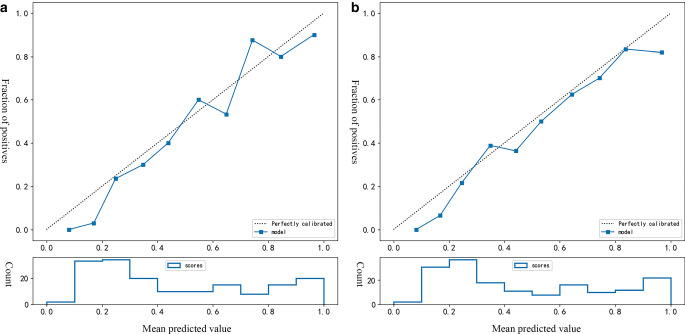
The calibration curves of integrated model in the training (**a**) and validation cohort (**b**). The closer the calibration curve is to the dotted line, the higher the calibration degree of the model

**Fig. 7:**
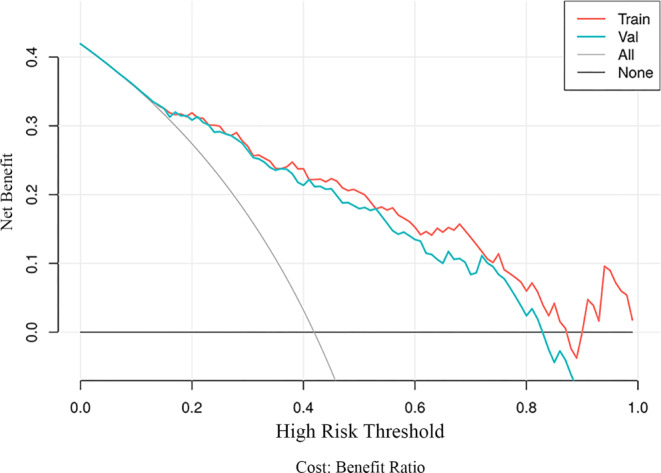
Decision curve analysis for the integrated model in the training and validation datasets. The y‑axis represented the net benefit, the x‑axis represented threshold probability. The gray curve line represented the assumption that all patients have symptomatic plaques, while the black horizontal line represented the assumption that no patients have symptomatic plaques. When the risk threshold range is 0.15–0.82, the model presents the benefit

The calibration curve of the combined model demonstrated a good fit between predicted and actual possibility of ischemic stroke in the training and validation cohorts (Fig. 6). The decision curves compare the net benefits at various threshold probabilities in the training and validation sets to demonstrate the clinical utility of the prediction model. The integrated model had an excellent overall net benefit when the risk threshold range was 0.15–0.82 (Fig. 7).

## Discussion

This study constructed an integrated predictive model that incorporated conventional clinical risk factors, plaque characteristics, and radiomics signature on CTA to identify symptomatic plaques, and validated its clinical utility using calibration and decision curves. The integrated model performed better than the conventional or radiomics model alone for plaque vulnerability assessment, which would assist in clinical management and improve patient outcomes.

The relationship between carotid plaque characteristics on CTA and ischemic stroke, such as plaque composition, vulnerability, and carotid artery tortuosity, has been extensively studied [4, 5, 9, 29]. According to the American Society of Neuroradiology and European Society of Cardiology guidelines, carotid plaque composition and the degree of stenosis are both factors that affect the risk of stroke [30, 31]. More attention should be focused on the comprehensive assessment of all risk factors rather than the degree of luminal stenosis alone. Our findings showed that plaque ulceration, carotid rim sign, and Hcy were independent risk factors. Plaque ulceration was speculated to be a risk factor for ipsilateral ischemic stroke [32]. A systematic review and meta-analysis found that plaque ulceration increased the risk of ipsilateral ischemia by 2.2 times [9]. There was a strong correlation between carotid rim sign and IPH, which was generally accepted as a strong predictor of cerebrovascular events [23, 33]. Nardi et al. found that IPH was often observed in mild carotid artery stenosis and suggested that IPH might associate with non-carotid artery occlusive stroke [34]. The plasma Hcy level would affect endothelial function, cell-cell adhesion, and oxidative response, which accelerates plaque formation and adds risk of acute cerebral infarction [35–38].

Different carotid plaque components present different CT attenuation values; however, due to the partial volume effect, it is difficult to visually differentiate the internal composition of plaques on CTA. Radiomics provides an objective, automated, and data-driven description of plaque features by measuring heterogeneity or shape that are mostly invisible to the human eye [15]. A proof-of-principle study found that radiomics was a feasible and reliable method to extract information beyond luminal stenosis in carotid CTA [39]. In our study, we chose six significant radiomic features and found that plaques with higher heterogeneity and lower strength features were more likely to be symptomatic, indicating that these plaques might contain more complicated components such as lipids or hemorrhage. Our results demonstrated that radiomic features of carotid plaques were strongly associated with ipsilateral ischemic stroke and could be used to predict plaque vulnerability. Radiomics signatures of carotid plaque on CTA may be a promising tool for identifying high-risk plaques and assessing stroke risk.

A recent case-control study of 24 patients demonstrated that CT texture characteristics could distinguish symptomatic and asymptomatic patients with carotid plaques [11]. Another study included 120 patients with moderate-to-severe carotid stenosis (> 50%).and revealed that the radiomics model improved the diagnostic performance of symptomatic plaques compared to conventional clinical assessment [20]. Our results were consistent with these findings that radiomic features could effectively identify symptomatic carotid plaques; however, Baradaran et al. found that there was also a high risk of ischemic stroke in patients with mild carotid stenosis [4]. In this study, we included patients with all degrees of atherosclerosis carotid stenosis (45.7% of patients in the symptomatic group with carotid stenosis < 50%). We further proposed a comprehensive model incorporating clinical risk factors, plaque features, and radiomic features, and achieved better performance than the conventional and radiomics models. Our work would add evidence to the literature which applied radiomics for plaque vulnerability assessment.

Our study has several limitations. First, we conducted a single-center retrospective study with a small sample size, and all enrolled patients underwent CTA examination using the same CT scanner and imaging protocol. We performed fivefold cross-validation and calculated a reliable estimate of diagnostic accuracy. Our results need further validation on external cohorts using data from different vendors and machines. Second, we included patients with large artery atherosclerosis stroke and excluded other etiologies as much as possible; however, we could not ascertain that all strokes were caused by the carotid atherosclerotic plaque. Third, there was inevitable subjectivity and variability when manually extracting carotid plaques. We removed radiomic features with interobserver and intraobserver ICC ≤ 0.8 to ensure the retained features were highly consistent and prevent subjective error. Future studies will be conducted on large-scale, prospective cohorts and integrate more clinical, imaging, and radiomics features to improve stroke risk stratification.

## Conclusion

The CTA-derived radiomics signature of carotid plaques could effectively identify symptomatic plaques. Radiomic features combined with conventional clinical and imaging parameters may be a useful tool for improving risk stratification and guiding clinical decision-making in patients with carotid atherosclerotic plaques.

### Supplementary Information

Table S1: Reproducibility of conventional features. Fig. S1: Reproducibility of radiomics features.
