# Supplementary material for: Radiomics Signatures of Carotid Plaque on Computed Tomography Angiography: An Approach to Identify Symptomatic Plaques
Source: Clin Neuroradiol. 2023 May 17;33(4):931–41. doi: 10.1007/s00062-023-01289-9 (PMC10654187; doi:10.1007/s00062-023-01289-9)
Supplement: Supplementary file 1 — Table S1: Reproducibility of conventional features. Fig. S1: Reproducibility of radiomics features. [file 62_2023_1289_MOESM1_ESM.pdf]

Supplementary Material

Article title:

Radiomics signatures of carotid plaque on computed tomography angiography: an approach to identify symptomatic plaques

Journal name:

Clinical Neuroradiology

Author names:

Jinglong Shi, Yu Sun, Jie Hou, Xiaogang Li, Jitao Fan, Libo Zhang, Rongrong Zhang, Hongrui You, Zhenguo Wang, Anxiaonan Zhang, Jianhua Zhang, Qiuyue Jin, Lianlian Zhao, Benqiang Yang

Corresponding author: Benqiang Yang

Affiliation:

Department of Radiology, General Hospital of Northern Theater Command, Shenyang, China

Key Laboratory of Cardiovascular Imaging and Research, Liaoning Province, China

E-mail: bqyang888@sina.com

Table S1 Reproducibility of conventional features.

Conventional features	Intra-observer	Inter-observer
CCTI	0.962	0.956
ICTI	0.960	0.932
Degree of stenosis	0.992	0.980
Total plaque thickness	0.997	0.990
Soft plaque thickness	0.998	0.994
Carotid plaque length	0.997	0.963
Plaque ulceration	1*	1*
Carotid rim sign	0.902*	0.815*
PR	0.933*	0.791*

Intra-class correlation coefficient (ICC) and Cohen's kappa coefficient were calculated for the intra- and inter-observer consistency. The intra- and inter-observer reproducibility of conventional features were substantial and excellent. *CCTI* common carotid tortuosity index, *ICTI* internal carotid tortuosity index, *PR* positive remodeling

* Indicated Cohen's kappa coefficient

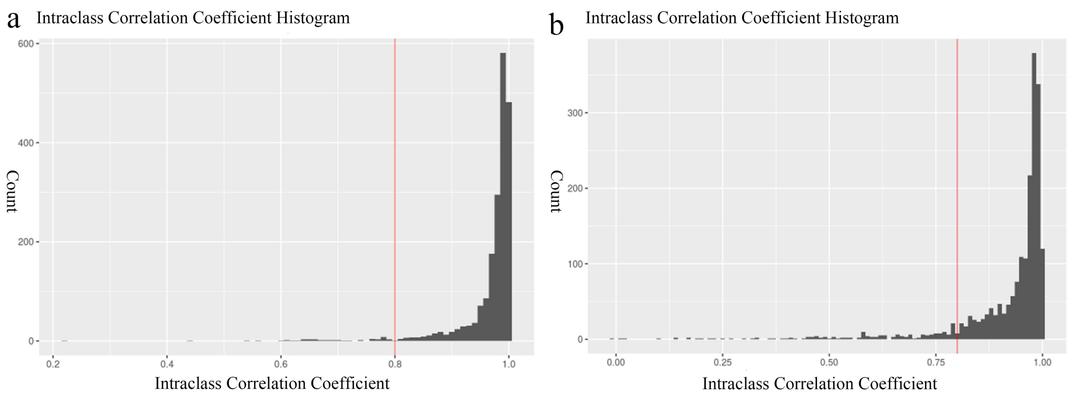

Fig. S1 Reproducibility of radiomics features.

Radiomics features with ICC ≤ 0.8 were removed by intra-observer (a, 49 features) and inter-observer (b, 182 features) agreement, and the remaining 1916 features were included in the analysis.
